# Association between Initial Fibrinogen Levels and the Need for Massive Transfusion in Emergency Department Patients with Primary Postpartum Hemorrhage: A Retrospective Study from a Single Center in Korea

**DOI:** 10.3390/jpm14040344

**Published:** 2024-03-26

**Authors:** Sungmin Park, Changhwan Sohn, Hyojeong Kwon, Sangmin Kim, Seungmok Ryoo, Shin Ahn, Dongwoo Seo, Wonyoung Kim

**Affiliations:** Department of Emergency Medicine, Asan Medical Center, University of Ulsan College of Medicine, Seoul 05505, Republic of Korea; d200659@amc.seoul.kr (S.P.); hyojeong1214@amc.seoul.kr (H.K.); d200226@amc.seoul.kr (S.K.); chrisryoo@amc.seoul.kr (S.R.); ans1023@amc.seoul.kr (S.A.); sdw@amc.seoul.kr (D.S.); d080593@amc.seoul.kr (W.K.)

**Keywords:** postpartum hemorrhage, blood transfusion, fibrinogen, predictive value of tests

## Abstract

Background: This study aimed to evaluate the association between initial fibrinogen levels and massive transfusion (MT) in emergency department (ED) patients with primary postpartum hemorrhage (PPH). Methods: This retrospective study was conducted in the ED of a university-affiliated, tertiary referral center from January 2004 to August 2023. Patients were divided into two groups: the MT group, which included those who received a transfusion of 10 or more units of packed red blood cells within the first 24 h, and the Non-MT group. Results: Out of the 364 patients included in the study, 97 (26.6%) required MT. Fibrinogen, shock index, and lactate were independently associated with MT (odds ratio [OR] 0.987; 95% confidence interval [CI] 0.983–0.991; *p* < 0.001, OR 7.277; 95% CI 1.856–28.535; *p* = 0.004, and OR 1.261; 95% CI 1.021–1.557; *p* = 0.031, respectively). The area under the receiver operating characteristic curve for fibrinogen, shock index, and lactate in predicting MT was 0.871 (95% CI 0.832–0.904; *p* < 0.001), 0.821 (95% CI 0.778–0.859; *p* < 0.001), and 0.784 (95% CI 0.738–0.825; *p* < 0.001), respectively. When the cutoff value of fibrinogen was 400 mg/dL, both the sensitivity and negative predictive values for predicting MT were 100.0%. When the cutoff value of fibrinogen was 100 mg/dL, the specificity and positive predictive values were 91.8% and 70.7%, respectively. Conclusion: The initial fibrinogen levels were independently associated with the need for MT in ED patients with primary PPH.

## 1. Introduction

Postpartum hemorrhage (PPH) is a significant contributor to maternal mortality and morbidity, highlighting the importance of prompt resuscitation [1]. Accurately calculating the amount of bleeding is crucial for timely initial resuscitation, including blood transfusion and fluid administration, but it is very challenging in actual clinical practice [2,3]. It is particularly challenging to accurately assess the amount of bleeding in patients with primary PPH who are transferred to the emergency department (ED) after delivery in other clinics or hospitals. The estimated amount of bleeding is likely to be highly inaccurate in such cases [4,5,6,7,8]. If there is significant bleeding, a substantial blood transfusion will be necessary to effectively resuscitate the patient. Therefore, it may be possible to identify predictors that can estimate the amount of bleeding based on the transfusion amount for real patients. Previous studies have shown an association between shock index, lactate levels, and the need for MT in ED patients with primary PPH [2,3,9]. However, the test performance of the shock index and lactate levels in predicting the need for MT was not sufficiently high [2,3,9].

Fibrinogen levels increase during pregnancy and decrease during PPH [10,11,12,13,14,15]. Several studies have identified an association between fibrinogen levels and the severity of PPH [16,17,18]. However, there are still few studies confirming the association between the initial fibrinogen levels and MT in ED patients with primary PPH [19]. We hypothesized that initial fibrinogen levels are associated with MT in ED patients with primary PPH and the cutoff value of the fibrinogen level indicating the need for MT would differ from the cutoff values reported in previous studies involving patients other than those in the ED.

Therefore, this study aimed to evaluate the association between initial fibrinogen levels and the need for MT in ED patients with primary PPH.

## 2. Materials and Methods

### 2.1. Study Design and Setting

This retrospective observational study was conducted in the ED of a 2800-bed university-affiliated tertiary referral center in South Korea. All patients diagnosed with primary PPH who underwent blood fibrinogen and lactate assessments between 1 January 2004 and 31 August 2023 were included. The study patients were initially identified through a hospital computer database system using a hospital discharge diagnosis of ‘postpartum hemorrhage’ [3]. Finally, patients with primary PPH were identified. Primary PPH was defined as excessive hemorrhage that required blood transfusion or fluid resuscitation within the first 24 h following delivery [3]. None of the patients included in this study were delivered at our hospital [3]. Instead, they were referred to our ED for the evaluation and management of primary PPH from other clinics or hospitals following delivery [3]. Patients were excluded if their fibrinogen or lactate assessments were not conducted upon admission to the ED, or if they received fresh frozen plasma before arriving at our ED, or if they had incomplete data.

During the study period, there were no specific guidelines or specialized teams for PPH treatment at our hospital. Actual treatment was carried out according to the judgment of the emergency medicine physicians and obstetricians responsible for the patient.

When patients with primary PPH visited our ED, a blood fibrinogen and lactate test were performed within minutes of their arrival. Fibrinogen levels were measured using the CA7000 (Sysmex Corporation, Kobe, Japan) (1 January 2003~1 November 2016), CA5100 (Sysmex Corporation, Kobe, Japan) (2 November 2016~3 July 2023), and CN6000 (Sysmex, Kobe, Japan) (4 July 2023~31 August 2023). During the study period, the reference values for all three devices used during the study period were 200–400 mg/dL. Blood lactate levels were measured in arterial or venous whole blood using a point-of-care blood gas analyzer (GEM Premier 3500 with iQM, Bedford, MA, USA) [3]. This analyzer can detect lactate levels ranging from 0.3 to 15.0 mM/L and provides results within 1 min [3].

The primary outcome of this study was the need for MT. MT was defined as the transfusion of 10 units or more of packed red blood cells within the first 24 h after the onset of PPH [2,3]. We calculated the amount of blood transfused before arrival at the ED and the amount of blood transfused after arrival at the ED (in the ED, intensive care unit (ICU), or general ward) to determine MT [3]. Patients were divided into two groups: the MT group, consisting of patients who required MT, and the Non-MT group, consisting of patients who did not require MT.

### 2.2. Data Collection

Data on the baseline and clinical characteristics of the patients were collected from electronic medical records. The collected data included the following: age, parity (primipara or multipara), type of delivery (vaginal delivery or Caesarean section), initial mental status, initial vital signs (systolic blood pressure, diastolic blood pressure, heart rate, and body temperature), initial laboratory findings (white blood cells, C-reactive protein, hemoglobin, hematocrit, platelets, prothrombin time, blood urea nitrogen, creatinine, and fibrinogen), amount of blood transfusion, and clinical outcomes (embolization, emergency hysterectomy, intensive care unit admission, in-hospital death, and length of hospital stay). Upon arrival at the ED, patients underwent triage, during which their initial mental status and vital signs were assessed. The AVPU (Alert/Verbal/Painful/Unresponsive) scale was used to assess the patient’s level of consciousness. The initial shock index, calculated by dividing the heart rate by the systolic blood pressure, was derived from the initial vital signs [2].

### 2.3. Statistical Analysis

Continuous variables were presented as medians and interquartile ranges, while categorical variables were presented as frequencies. Categorical variables were compared using either Pearson’s χ^2^ test or Fisher’s exact test. Continuous variables were initially assessed for normal distribution using the Kolmogorov–Smirnov test and then compared using either the Student’s *t*-test or the Mann–Whitney U-test, depending on their distribution.

Binary logistic regression analysis was used to identify variables independently associated with the need for MT, and all variables that were statistically significant in univariate analysis were included. Stepwise modeling was used to identify variables for inclusion in the final model. The results of the binary logistic regression analyses were presented as odds ratios (ORs) and 95% confidence intervals (CIs).

The area under the curve (AUC) of the receiver operating characteristic (ROC) curve was used to validate the discriminatory power of the initial fibrinogen level in predicting the need for MT. The sensitivity, specificity, positive predictive value, and negative predictive value were used to assess the test’s performance in predicting the need for MT based on the initial fibrinogen level.

A two-sided *p* value less than 0.05 was considered statistically significant. The statistical analyses were performed using PASW Statistics for Windows version 23.0 (IBM Corp., Armonk, NY, USA) and MedCalc^®^ Statistical Software version 20.116 (MedCalc Software Ltd., Ostend, Belgium). The actual power of this study was calculated by statistical power tool G*Power (version 3.1.9.7, HHU-Düsseldorf, Germany).

## 3. Results

### 3.1. Baseline and Clinical Characteristics

Out of a total of 612 patients with primary PPH who were screened during the study period, 153 patients without an assessment of initial blood fibrinogen and lactate levels, 90 patients who received fresh frozen plasma transfusion before arriving at our ED, and 5 patients with incomplete data were excluded, leaving 364 patients for the final analysis. Out of the 364 patients finally included, 97 (26.6%) required MT. The actual power of this study was 0.99 (effect size 0.5, alpha error probability 0.05).

Baseline and clinical characteristics of patients categorized according to the need for MT are summarized in Table 1. There were no significant differences in age, parity, delivery method, causes of PPH, and body temperature between the two groups. However, there was a significant difference in the initial mental status between the two groups (*p* < 0.001). The MT group had significantly lower systolic and diastolic blood pressure and a higher heart rate upon arrival at the ED compared to the Non-MT group (*p* < 0.001). The initial shock index upon arrival at the ED was significantly higher in the MT group than in the Non-MT group (1.1 vs. 0.8, *p* < 0.001).

A comparison of baseline and clinical characteristics in patients who received vaginal delivery and patients who underwent Caesarean section is summarized in Table S1. There was no statistically significant difference in age, parity, initial mental status, and initial vital signs between the two groups. However, there was a statistically significant difference in the causes of PPH (*p* = 0.013).

A comparison of baseline and clinical characteristics of patients according to the causes of PPH is summarized in Table S2. There was no statistically significant difference in age, parity, initial mental status, and initial vital signs between the uterine atony group and others groups.

### 3.2. Comparison of Initial Laboratory Findings, Management, and Clinical Outcomes between the Massive Transfusion (MT) Group and the Non-Massive Transfusion (Non-MT) Group

The initial laboratory findings, management, and clinical outcomes based on the need for MT are compared in Table 2. The MT group had significantly higher lactate levels, lower hemoglobin, hematocrit, and platelet counts, prolonged prothrombin time, required more units of blood components, used more vasopressors and tranexamic acid, and underwent more embolization, hysterectomy (*p* = 0.005), hematoma evaluation, and required more intensive care unit care compared to the Non-MT group (*p* < 0.001 except for hysterectomy). In addition, the MT group had longer hospital stays and were more likely to require ICU admission compared to the Non-MT group (*p* < 0.001). In-hospital mortality was higher in the MT group than in the Non-MT group (*p* = 0.018).

A comparison of the initial laboratory findings, management, and clinical outcomes in patients who received vaginal delivery and patients who underwent Caesarean section is summarized in Table S3. There was no statistically significant difference in initial laboratory findings, including fibrinogen levels, management, and clinical outcomes between the two groups.

A comparison of the initial laboratory findings, management, and clinical outcomes according to the causes of PPH is summarized in Table S4. There was no statistically significant difference in initial laboratory findings, including fibrinogen levels, management, and clinical outcomes between the uterine atony group and others groups.

### 3.3. Predictive Performance of Initial Fibrinogen Levels in Predicting the Need for Massive Transfusion (MT)

**Figure 1:**
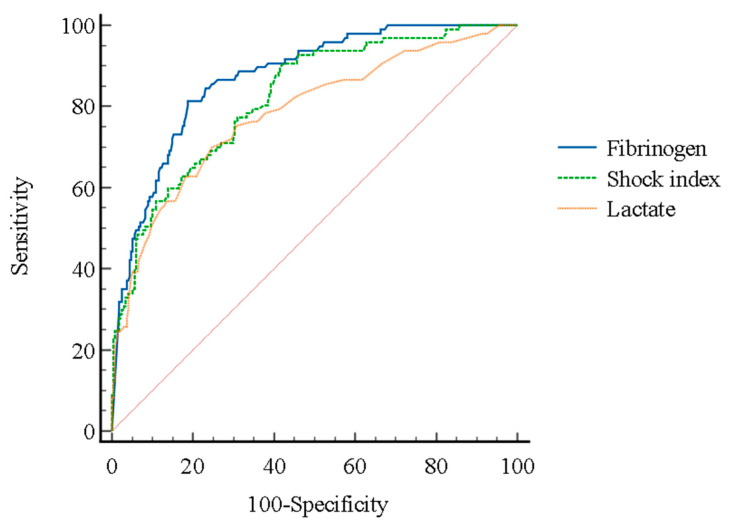
The receiver operating characteristic (ROC) curves of fibrinogen, shock index, and lactate in predicting the need for massive transfusion in emergency department patients with primary postpartum hemorrhage.

The fibrinogen, shock index, and lactate were independently associated with MT (OR, 0.987; 95% CI, 0.983–0.991; *p* < 0.001, 7.277; 95% CI, 1.856–28.535; *p* = 0.004 and 1.261; 95% CI, 1.021–1.557; *p* = 0.031, respectively) (Table 3). The area under the ROC curve (AUC) for fibrinogen, shock index, and lactate in predicting MT was 0.871 (95% CI, 0.832–0.904; *p* < 0.001), 0.821 (95% CI, 0.778–0.859; *p* < 0.001), and 0.784 (95% CI, 0.738–0.825; *p* < 0.001), respectively (Table 4, Figure 1). The frequency of MT increased as initial fibrinogen levels decreased (Table 5). When the fibrinogen cutoff value was set at 400 mg/dL, both the sensitivity and negative predictive values for predicting MT were 100.0% (Table 6). When the fibrinogen cutoff value was set at 100 mg/dL, the specificity and positive predictive values were 91.8% and 70.7%, respectively.

## 4. Discussion

This study evaluated the association between initial fibrinogen levels upon admission to the ED and the need for MT in ED patients with primary PPH. The study identified that the initial levels of fibrinogen were independently associated with MT. Fibrinogen levels of 400 mg/dL or higher were a significant negative predictor of MT, while fibrinogen levels of less than 100 mg/dL were a significant positive predictor of MT. Through this study, we identified that, in addition to the previously known association between the severity of PPH and fibrinogen levels in patients outside the ED, the initial fibrinogen level was also correlated with the need for MT in ED patients with primary PPH. These results suggest that it is possible to predict whether a patient will require MT by measuring the initial fibrinogen level in the ED setting.

Fibrinogen is a vital plasma protein coagulation factor that plays a critical role in hemostasis and thrombosis [20]. It is synthesized in hepatocytes and serves as a precursor for fibrin, while also activating platelets through binding to activated platelets [20]. The normal range of fibrinogen in non-pregnant women is from 2.3 to 5.0 g/L [21]. However, as the number of trimesters increases during pregnancy, fibrinogen levels also increase. The normal range of fibrinogen is 2.4–5.1 g/L during the first trimester, 2.9–5.4 g/L during the second trimester, and 3.7–6.2 g/L during the third trimester [21]. After delivery, fibrinogen levels decrease, and this reduction persists during the early postpartum period. When PPH occurs, fibrinogen levels rapidly decrease due to the loss of fibrinogen from bleeding, an increase in fibrinolytic activity, and hemodilution from fluid resuscitation [22]. Therefore, in patients with PPH, the fibrinogen level may indicate the severity of the hemorrhage.

Several previous studies have evaluated the association between fibrinogen levels and the severity of PPH [16,17,18]. In a study by Charbit et al., severe PPH was defined as a peripartum decrease in hemoglobin of 4 g/dL or more (with the last hemoglobin value before delivery considered as the reference), transfusion of at least 4 units of red blood cells, hemostatic intervention (such as angiographic embolization, surgical arterial ligation, or hysterectomy), or death [16]. The study found that a reduction in fibrinogen levels during the early stage of PPH was associated with severe PPH [16]. In a study by Cortet et al., severe PPH was defined as a decrease in peripartum hemoglobin level of ≥40 g/L, transfusion of concentrated red cells, arterial embolization, emergency surgery, admission to intensive care, or death [17]. The fibrinogen level at the time of PPH diagnosis was found to be associated with severe PPH [17]. These two studies examined the fibrinogen levels at the time of diagnosis of PPH at the hospital where the delivery took place [16,17]. However, in the ED setting, patients may be transferred to another hospital’s ED due to uncontrolled PPH, despite being diagnosed and treated at the hospital where the delivery occurred. This suggests that the severity of their condition may be more significant than that of the patients in the previous two studies. The primary outcome in this study differed from that of the previous two studies, making it difficult to directly compare the study results. However, when considering the transfusion aspect, Charbit et al. reported that 7.0% of patients required transfusion of more than 4 units of packed red blood cells, while Cortet et al. reported that 18.4% of patients required transfusion of more than 1 unit [16,17]. In this study, 26.6% of all patients required a transfusion of 10 units or more of packed red blood cells within the first 24 h. Based on these results, it can be concluded that the severity of PPH in this study is higher than in the previous two studies.

Asami Okada et al. examined the association between fibrinogen and lactate levels and the need for MT, defined as the administration of more than 10 units of packed red blood cells within the first 24 h, in ED patients with PPH [19]. The crude odds ratio (OR) for fibrinogen levels was 0.98 (95% CI, 0.97–0.99), and for lactate levels, it was 1.62 (95% CI, 1.08–3.02). The area under the curve (AUC) was 0.814 for fibrinogen levels and 0.734 for lactate levels [19]. The optimal cutoff values for fibrinogen and lactate levels were 211 mg/dL and 4 mmol/L, respectively [19]. The shock index was found to have no association with MT. However, the sample size was too small, with only 31 patients in total, and multivariate analysis was not performed as a result. This study confirmed the association between fibrinogen levels and MT by using a substantial sample size and conducting multivariate analysis to overcome the limitations of the previous study [19]. Furthermore, it was confirmed that the shock index was associated with the need for MT, consistent with previous studies [2,3,9]. These findings may provide significant evidence in this topic area. The frequency of MT increased as initial fibrinogen levels decreased. When the cutoff value of the initial fibrinogen level was set at 400 mg/dL, no patients required MT, resulting in both 100% sensitivity and negative predictive value. When the cutoff value of the initial fibrinogen level was set at 100 mg/dL, MT was required in 70.7% of patients, with a specificity of 91.8% and a positive predictive value of 70.7%. These findings suggest that initial fibrinogen levels can be used to determine whether MT is necessary for ED patients with primary PPH.

This study has several limitations. First, it is important to note that this study was conducted retrospectively, which may introduce potential biases and confounding factors into our findings, as commonly discussed in the literature. Prospective, validated study is essential to validate these findings. Second, our study exclusively involved patients from a single medical center, thus limiting the generalizability of our findings to other institutions or patient cohorts. Third, because the study period lasted for up to 20 years, the same treatment may not have been consistently provided to patients due to advances in treatment during that time, which may have affected the clinical results. Additionally, during the study period, our institution did not have specific transfusion guidelines for ED patients with primary PPH. Consequently, the decision of whether to administer blood transfusions, as well as the type and quantity of blood to be transfused, was entirely at the discretion of the emergency physicians or obstetricians responsible for the patient’s care [3]. Fourth, the administration of tranexamic acid may have affected fibrinogen levels and subsequent clinical outcomes. In this study, blood samples for the fibrinogen test were taken before the administration of tranexamic acid, thereby allowing for the exclusion of any potential impact of tranexamic acid on fibrinogen levels. Nonetheless, it cannot be dismissed that the administration of tranexamic acid may have impacted the clinical outcomes. Fifth, during the study period, there were a total of three equipment changes. Even though the reference range for fibrinogen levels was consistent across all three pieces of equipment (200–400 mg/dL), variations in measured values may occur due to equipment differences. Sixth, changes in fibrinogen levels may occur as a result of blood transfusion or fluid administration. Patients who received fresh frozen plasma before fibrinogen level measurement were excluded from the study to eliminate any potential changes caused by blood transfusion. However, all the patients included in this study were transferred from other hospitals, which made it impossible to calculate the volume of fluid administered before measuring the fibrinogen level. As a result, there may have been changes in fibrinogen levels as a result of fluid administration. Seventh, serial measurements of fibrinogen levels can provide more precise insights for predicting the need for MT. However, this was not reflected in this study. Finally, the study period is extensive, spanning approximately 20 years. During the extended study period, there may have been changes in the criteria for hemoglobin levels for blood transfusion. Additionally, patient blood management during pregnancy, such as taking iron supplements, may vary. Depending on whether patients take iron supplements during pregnancy, hemoglobin levels may be affected. However, due to the retrospective nature of this study, it was not possible to investigate this.

## 5. Conclusions

The initial fibrinogen levels upon admission to the ED were independently associated with the need for MT in ED patients with primary PPH. In addition, fibrinogen levels of 400 mg/dL or higher were a significant negative predictor of the need for MT, while fibrinogen levels of less than 100 mg/dL were a significant positive predictor of the need for MT. These findings suggest that initial fibrinogen levels may serve as a biomarker indicating the need for MT in ED patients with primary PPH.

## Figures and Tables

**Table 1:** Comparisons of baseline and clinical characteristics in emergency department patients with primary postpartum hemorrhage, based on the need for massive transfusion.

Variables	All Patients (*n* = 364)	MT Group (*n* = 97)	Non-MT Group (*n* = 267)	*p* Value
Age, years	33.0 (30.0–35.0)	34.0 (31.0–36.0)	33.0 (30.0–35.0)	0.055
Parity				0.47
Primipara	225 (61.8)	57 (58.8)	168 (62.9)	
Multipara	139 (38.2)	40 (41.2)	99 (37.1)	
Delivery type				0.406
Vaginal delivery	245 (67.3)	62 (63.9)	183 (68.5)	
Caesarean section	119 (32.7)	35 (36.1)	84 (31.5)	
Causes				0.689
Uterine atony	210 (57.7)	56 (57.7)	154 (57.7)	
Uterine rupture	9 (2.5)	6 (6.2)	3 (1.1)	
Uterine inversion	1 (0.3)	0 (0.0)	1 (0.4)	
Trauma to the genital tract	60 (16.5)	16 (16.5)	44 (16.5)	
Retained placenta	11 (3.0)	0 (0.0)	11 (4.1)	
Low lying placenta	4 (1.1)	2 (2.1)	2 (0.7)	
Placenta abruptio	5 (1.4)	3 (3.1)	2 (0.7)	
Placenta accreta	15 (4.1)	0 (0.0)	15 (5.6)	
Placenta previa	4 (1.1)	1 (1.0)	3 (1.1)	
Undetermined	45 (12.4)	12 (13.4)	32 (12.0)	
Initial mental status				<0.001
Alert	346 (95.1)	82 (84.5)	264 (98.9)	
Verbal	10 (2.7)	8 (8.2)	2 (0.7)	
Painful	4 (1.1)	3 (3.1)	1 (0.4)	
Unresponsive	4 (1.1)	4 (4.1)	0 (0.0)	
Initial vital signs				
Systolic blood pressure, mmHg	114.0 (97.0–127.0)	98.0 (80.0–120.0)	116.0 (104.0–129.0)	<0.001
Diastolic blood pressure, mmHg	69.0 (59.0–80.0)	61.5 (50.5–72.8)	72.0 (62.0–81.0)	<0.001
Heart rate, beats/min	96.0 (84.0–110.0)	114.0 (98.5–135.5)	91.0 (81.0–102.0)	<0.001
Body temperature, ℃	37.2 (36.6–37.8)	37.0 (36.5–37.6)	37.2 (36.6–37.9)	0.663
Shock index	0.84 (0.71–1.08)	1.19 (0.91–1.56)	0.77 (0.66–0.95)	<0.001

Values are expressed as the median (interquartile range) or as a number (%).

**Table 2:** Comparisons of initial laboratory findings, management, and clinical outcomes in emergency department patients with primary postpartum hemorrhage, based on the need for massive transfusion.

Variables	All Patients (*n* = 364)	MT Group (*n* = 97)	Non-MT Group (*n* = 267)	*p* Value
Initial laboratory findings				
White blood cells, ×10^3^/µL	17.1 (13.7–21.2)	17.4 (13.6–23.5)	17.0 (13.8–20.9)	0.327
C-reactive protein, mg/dL	0.4 (0.2–0.8)	0.4 (0.2–0.6)	0.5 (0.2–0.9)	0.102
Hemoglobin, g/dL	9.6 (8.1–10.9)	7.9 (5.7–9.9)	9.9 (8.7–11.2)	<0.001
Hematocrit, %	29.2 (25.1–32.8)	24.4 (17.9–29.4)	30.3 (26.6–33.5)	<0.001
Platelets, ×10^3^/µL	152.0 (118.0–191.0)	120.0 (90.0–155.5)	163.0 (134.0–198.0)	<0.001
Prothrombin time (INR)	1.1 (1.0–1.3)	1.6 (1.2–2.5)	1.1 (1.0–1.2)	<0.001
Blood urea nitrogen, mg/dL	7.0 (6.0–10.0)	9.0 (6.3–11.0)	7.0 (6.0–9.0)	<0.001
Creatinine, mg/dL	0.6 (0.5–0.7)	0.7 (0.6–0.9)	0.6 (0.5–0.7)	<0.001
Fibrinogen, mg/dL	208.0 (121.3–307.3)	90.0 (25.0–162.0)	252.0 (183.0–333.0)	<0.001
Lactate, mmol/L	2.5 (1.8–3.88)	4.2 (2.9–5.8)		<0.001
Management				
Blood transfusion, units	327 (89.8)	97 (100.0)	230 (86.1)	<0.001
pRBCs before ED arrival	0.0 (0.0–2.0)	2.0 (0.0–4.0)	0.0 (0.0–2.0)	<0.001
Total pRBCs	5.0 (2.0–10.0)	14.0 (12.0–20.0)	3.0 (2.0–5.0)	<0.001
FFP before ED arrival	0.0 (0.0–0.0)	0.0 (0.0–0.0)	0.0 (0.0–0.0)	1
Total FFP	3.0 (0.0–6.0)	10.0 (6.0–13.0)	2.0 (0.0–3.0)	<0.001
PCs before ED arrival	0.0 (0.0–0.0)	0.0 (0.0–0.0)	0.0 (0.0–0.0)	0.547
Total PCs	0.0 (0.0–8.0)	10.0 (8.0–18.5)	0.0 (0.0–0.0)	<0.001
Uterotonics use	341 (93.7)	94 (96.9)	247 (92.5)	0.127
Vasopressor use	19 (5.2)	18 (18.6)	1 (0.4)	<0.001
Tranexamic acid use	61 (16.8)	27 (27.8)	34 (12.7)	<0.001
Embolization	183 (50.3)	84 (86.6)	99 (37.1)	<0.001
Hysterectomy	4 (1.1)	4 (4.1)	0 (0.0)	0.005
Hematoma evacuation	23 (6.3)	15 (15.5)	8 (3.0)	<0.001
Intensive care unit care	30 (8.2)	26 (26.8)	4 (1.5)	<0.001
Clinical outcome				
In-hospital mortality	3 (0.8)	3 (3.1)	0 (0.0)	0.018
Days of hospitalization	2.0 (1.0–4.0)	4.0 (3.0–6.0)	2.0 (1.0–3.0)	<0.001

Values are expressed as the median (interquartile range) or as a number (%). INR, international normalized ratio; pRBCs, packed red blood cells; ED, emergency department; FFP, fresh frozen plasma; PCs, platelet concentrates.

**Table 3:** Multivariate logistic regression analysis identifying the independent factors associated with the need for massive transfusion in emergency department patients with primary postpartum hemorrhage.

Variables	Odds Ratio	95% Confidence Interval	*p* Value
Shock index	7.277	1.856–28.535	0.004
Lactate	1.261	1.021–1.557	0.031
Fibrinogen	0.987	0.983–0.991	<0.001

**Table 4:** Comparisons of the area under the receiver operating characteristic (ROC) curves for fibrinogen, shock index, and lactate in predicting the need for massive transfusion in emergency department patients with primary postpartum hemorrhage.

Variables	AUC	95% Confidence Interval	*p* Value	Cutoff Value	Sensitivity (%)	Specificity (%)
Fibrinogen	0.871	0.832–0.904	<0.001	167	81.4	81.3
Shock index	0.821	0.778–0.859	<0.001	0.805	90.7	57.7
Lactate	0.784	0.738–0.825	<0.001	3.1	70.1	75.3

**Table 5:** Comparisons of the frequency of massive transfusion based on initial fibrinogen levels in emergency department patients with primary postpartum hemorrhage.

Variables	MT Group *n* (%)	Non-MT Group *n* (%)	*p* Value
			<0.001
Fibrinogen > 400 mg/dL, *n* = 20	0 (0.0)	20 (100.0)	
300 < Fibrinogen ≤ 400 mg/dL, *n* = 76	2 (2.6)	74 (97.4)	
200 < Fibrinogen ≤ 300 mg/dL, *n* = 98	9 (9.2)	89 (90.8)	
100 < Fibrinogen ≤ 200 mg/dL, *n* = 95	33 (34.7)	62 (65.3)	
Fibrinogen ≤ 100 mg/dL, *n* = 75	53 (70.7)	22 (29.3)	

**Table 6:** Test performance of initial fibrinogen levels in predicting the need for massive transfusion in emergency department patients with primary postpartum hemorrhage.

Cutoff Value (mg/dL)	Sensitivity (%) (95% CI)	Specificity (%) (95% CI)	PPV (%) (95% CI)	NPV (%) (95% CI)
400	100.0 (96.3–100.0)	7.5 (4.6–11.3)	28.2 (27.5–28.9)	100.0 (83.2–100.0)
300	97.9 (92.8–99.8)	35.2 (29.5–41.3)	35.5 (33.4–37.6)	97.9 (92.2–99.5)
200	88.7 (80.6–94.2)	68.5 (62.6–74.1)	50.6 (45.8–55.3)	94.3 (90.5–96.7)
100	54.6 (44.2–64.8)	91.8 (87.8–94.8)	70.7 (60.8–78.9)	84.8 (81.7–87.4)
167 *	80.4 (71.1–87.8)	81.3 (76.1–85.8)	60.9 (54.4–67.1)	92.0 (88.4–94.5)

PPV, positive predictive value; NPV, negative predictive value; CI, confidential interval. * The cutoff value is determined as the point on the receiver operating characteristic curve that maximizes the Youden index.

## Data Availability

Data are contained within the article.

## Supplementary Materials

The following supporting information can be downloaded at: https://www.mdpi.com/article/10.3390/jpm14040344/s1. Supplementary Material Table S1: Comparisons of baseline and clinical characteristics in emergency department patients with primary postpartum hemorrhage, based on the delivery type; Supplementary Material Table S2: Comparisons of initial laboratory findings, management, and clinical outcomes in emergency department patients with primary postpartum hemorrhage, based on the delivery type; Supplementary Material Table S3: Comparisons of baseline and clinical characteristics in emergency department patients with primary postpartum hemorrhage, based on the causes of postpartum hemorrhage; Supplementary Material Table S4: Comparisons of initial laboratory findings, management, and clinical outcomes in emergency department patients with primary postpartum hemorrhage, based on the causes of postpartum hemorrhage.
