# Supplementary material for: Association between Initial Fibrinogen Levels and the Need for Massive Transfusion in Emergency Department Patients with Primary Postpartum Hemorrhage: A Retrospective Study from a Single Center in Korea
Source: J Pers Med. 2024 Mar 26;14(4):344. doi: 10.3390/jpm14040344 (PMC11050853; doi:10.3390/jpm14040344)
Supplement: Supplementary file 1 [file jpm-14-00344-s001.zip › jpm-2886375-supplementary.pdf]

Table S1. Comparisons of baseline and clinical characteristics in emergency department patients with primary postpartum hemorrhage, based on the delivery type.

Variables	All patients (n=364)	Vaginal delivery group (n=245)	Caesarean section group (n=119)	*p* Value
Age, years	33.0 (30.0-35.0)	33.0 (30.0-35.0)	33.0 (30.0-36.0)	0.210
Parity				0.740
Primipara	225 (61.8)	150 (61.2)	75 (63.0)	
Multipara	139 (38.2)	95 (38.8)	44 (37.0)	
Causes				0.013
Uterine atony	210 (57.7)	138 (56.3)	72 (60.5)	
Uterine rupture	9 (2.5)	2 (0.8)	7 (5.9)	
Uterine inversion	1 (0.3)	1 (0.4)	0 (0.0)	
Trauma to the genital tract	60 (16.5)	57 (23.3)	3 (2.5)	
Retained placenta	11 (3.0)	8 (3.3)	3 (2.5)	
Low lying placenta	4 (1.1)	3 (1.2)	1 (0.8)	
Placenta abruptio	5 (1.4)	0 (0.0)	5 (4.2)	
Placenta accreta	15 (4.1)	10 (4.1)	5 (4.2)	
Placenta previa	4 (1.1)	1 (0.4)	3 (2.5)	
Undetermined	45 (12.4)	25 (10.2)	20 (16.8)	
Initial mental status				0.742
Alert	346 (95.1)	233 (95.1)	113 (95.0)	
Verbal	10 (2.7)	8 (3.3)	2 (1.7)	
Painful	4 (1.1)	1 (0.4)	3 (2.5)	
Unresponsive	4 (1.1)	3 (1.2)	1 (0.8)	
Initial vital signs				
Systolic blood pressure, mmHg	114.0 (97.0-127.0)	112.0 (96.0-125.0)	116.0 (102.0-134.0)	0.053
Diastolic blood pressure, mmHg	69.0 (59.0-80.0)	68.0 (57.0-79.0)	71.0 (63.0-80.0)	0.080
Heart rate, beats/min	96.0 (84.0-110.0)	96.0 (84.0-109.5)	94.0 (81.0-114.0)	0.674
Body temperature, °C	37.2 (36.6-37.8)	37.2 (36.6-37.9)	37.1 (36.5-37.7)	0.189
Shock index	0.84 (0.71-1.08)	0.87 (0.72-1.08)	0.81 (0.66-1.08)	0.111

Values are expressed as the median (interquartile range) or as a number (%).

Table S2. Comparisons of baseline and clinical characteristics in emergency department patients with primary postpartum hemorrhage, based on the causes of postpartum hemorrhage.

Variables	All patients (n=319)	Uterine atony group (n=210)	Others group (n=109)	*p* Value
Age, years	33.0 (30.0-35.0)	33.0 (30.0-35.0)	33.0 (30.0-35.3)	0.855
Parity				0.879
Primipara	195 (61.1)	129 (61.4)	66 (60.6)	
Multipara	139 (38.2)	95 (38.8)	44 (37.0)	
Initial mental status				0.742
Alert	304 (95.3)	198 (94.3)	106 (97.2)	
Verbal	9 (2.8)	7 (3.3)	2 (1.8)	
Painful	3 (0.9)	2 (1.0)	1 (0.9)	
Unresponsive	3 (0.9)	3 (1.4)	0 (0.0)	
Initial vital signs				
Systolic blood pressure, mmHg	113.0 (97.0-127.0)	112.0 (96.0-125.0)	111.5 (96.0-125.3)	0.164
Diastolic blood pressure, mmHg	69.5 (59.0-80.0)	68.0 (57.0-79.0)	68.0 (57.5-78.0)	0.053
Heart rate, beats/min	96.0 (84.0-111.0)	96.0 (84.0-109.5)	97.0 (83.8-111.0)	0.902
Body temperature, ℃	37.2 (36.6-37.9)	37.2 (36.6-37.9)	37.3 (36.6-37.9)	0.065
Shock index	0.86 (0.72-1.08)	0.87 (0.72-1.08)	0.87 (0.71-1.11)	0.615

Values are expressed as the median (interquartile range) or as a number (%).

Table S3. Comparisons of initial laboratory findings, management, and clinical outcomes in emergency department patients with primary postpartum hemorrhage, based on the delivery type.

Variables	All patients (n=364)	Vaginal delivery group (n=245)	Caesarean section group (n=119)	*p* Value
Initial laboratory findings				
White blood cells, x10^3/μL	17.1 (13.7-21.2)	17.5 (14.4-21.8)	16.4 (12.4-20.8)	0.055
C-reactive protein, mg/dL	0.4 (0.2-0.8)	0.5 (0.3-0.8)	0.4 (0.2-0.9)	0.213
Hemoglobin, g/dL	9.6 (8.1-10.9)	9.7 (8.3-11.0)	9.2 (7.9-10.7)	0.137
Hematocrit, %	29.2 (25.1-32.8)	29.7 (25.3-33.0)	28.5 (24.3-32.4)	0.156
Platelets, x10^3/μL	152.0 (118.0-191.0)	153.0 (119.5-191.5)	151.0 (117.0-187.0)	0.416
Prothrombin time (INR)	1.1 (1.0-1.3)	1.1 (1.0-1.3)	1.1 (1.1-1.4)	0.081
Blood urea nitrogen, mg/dL	7.0 (6.0-10.0)	7.0 (6.0-10.0)	8.0 (6.0-10.0)	0.187

Creatinine, mg/dL	0.6 (0.5-0.7)	0.6 (0.5-0.7)	0.6 (0.5-0.7)	0.617
Fibrinogen, mg/dL	208.0 (121.3-307.3)	217.0 (128.0-315.0)	192.0 (109.0-272.0)	0.086
Lactate, mmol/L	2.5 (1.8-3.9)	2.6 (1.8-4.0)	2.3 (1.8-3.7)	0.244
Management				
Blood transfusion, units	327 (89.8)	109 (91.6)	218 (89.0)	0.438
pRBCs before ED arrival	0.0 (0.0-2.0)	0.0 (0.0-2.0)	1.0 (0.0-2.0)	0.001
Total pRBCs	5.0 (2.0-10.0)	5.0 (2.0-10.0)	5.0 (2.0-12.0)	0.723
FFP before ED arrival	0.0 (0.0-0.0)	0.0 (0.0-0.0)	0.0 (0.0-0.0)	1.000
Total FFP	3.0 (0.0-6.0)	2.0 (0.0-5.5)	3.0 (0.0-7.0)	0.642
PCs before ED arrival	0.0 (0.0-0.0)	0.0 (0.0-0.0)	0.0 (0.0-0.0)	0.151
Total PCs	0.0 (0.0-8.0)	0.0 (0.0-8.0)	0.0 (0.0-8.0)	0.537
Massive transfusion	97 (26.6)	35 (29.4)	62 (25.3)	0.406
Uterotonics use	341 (93.7)	114 (95.8)	227 (92.7)	0.247
Vasopressor use	19 (5.2)	8 (6.7)	11 (4.5)	0.369
Tranexamic acid use	61 (16.8)	26 (21.8)	35 (14.3)	0.070
Embolization	183 (50.3)	57 (47.9)	126 (51.4)	0.528
Hysterectomy	4 (1.1)	2 (1.7)	2 (0.8)	0.600
Hematoma evacuation	23 (6.3)	12 (10.1)	11 (4.5)	0.040
Intensive care unit care	30 (8.2)	11 (9.2)	19 (7.8)	0.628
Clinical outcome				
In-hospital mortality	3 (0.8)	1 (0.8)	2 (0.8)	1.000
Days of hospitalization	2.0 (1.0-4.0)	2.0 (1.0-3.0)	2.0 (2.0-5.0)	0.068

Values are expressed as the median (interquartile range) or as a number (%).
INR, international normalized ratio; pRBCs, packed red blood cells; ED, emergency department; FFP, fresh frozen plasma; PCs, platelet concentrates.

Table S4. Comparisons of initial laboratory findings, management, and clinical outcomes in emergency department patients with primary postpartum hemorrhage, based on the causes of postpartum hemorrhage.

Variables	All patients (n=319)	Uterine atony group (n=210)	Others group (n=109)	*p* Value
Initial laboratory findings				
White blood cells, x10^3/μL	17.2 (13.7-21.2)	17.5 (14.4-21.8)	17.1 (13.6-21.0)	0.371
C-reactive protein, mg/dL	0.4 (0.2-0.8)	0.5 (0.3-0.8)	0.4 (0.2-0.8)	0.415
Hemoglobin, g/dL	9.6 (8.1-11.0)	9.7 (8.3-11.0)	9.5 (8.0-10.9)	0.291
Hematocrit, %	29.3 (25.2-33.0)	29.7 (25.3-33.0)	29.1 (24.6-33.0)	0.278
Platelets, x10^3/μL	149.0 (116.0-191.0)	153.0 (119.5-191.5)	146.5 (110.5-192.0)	0.228
Prothrombin time (INR)	1.1 (1.0-1.3)	1.1 (1.0-1.3)	1.1 (1.1-1.4)	0.090
Blood urea nitrogen, mg/dL	7.0 (6.0-9.0)	7.0 (6.0-10.0)	7.0 (6.0-9.0)	0.163
Creatinine, mg/dL	0.6 (0.5-0.7)	0.6 (0.5-0.7)	0.6 (0.5-0.7)	0.706
Fibrinogen, mg/Dl	206.0 (118.0-305.0)	217.0 (128.0-315.0)	203.0 (104.8-302.5)	0.313
Lactate, mmol/L	2.5 (1.8-3.8)	2.5 (1.8-4.0)	2.4 (1.8-3.7)	0.670
Management				
Blood transfusion, units	285 (89.3)	183 (87.1)	102 (93.6)	0.077
pRBCs before ED arrival	0.0 (0.0-2.0)	0.0 (0.0-2.0)	0.0 (0.0-2.0)	0.847
Total pRBCs	5.0 (2.0-10.0)	5.0 (2.0-10.0)	5.0 (2.0-11.0)	0.472
FFP before ED arrival	0.0 (0.0-0.0)	0.0 (0.0-0.0)	0.0 (0.0-0.0)	1.000
Total FFP	3.0 (0.0-6.0)	2.0 (0.0-5.5)	2.0 (0.0-6.3)	0.674
PCs before ED arrival	0.0 (0.0-0.0)	0.0 (0.0-0.0)	0.0 (0.0-0.0)	1.000
Total PCs	0.0 (0.0-8.0)	0.0 (0.0-8.0)	0.0 (0.0-8.0)	0.497
Massive transfusion	84 (26.3)	56 (26.7)	28 (25.7)	0.851
Uterotonics use	302 (94.7)	206 (98.1)	96 (88.1)	<0.001
Vasopressor use	16 (5.0)	13 (6.2)	3 (2.8)	0.182
Tranexamic acid use	52 (16.3)	34 (16.2)	18 (16.5)	0.941
Embolization	164 (51.4)	116 (55.2)	48 (44.0)	0.058
Hysterectomy	3 (0.9)	3 (1.4)	0 (0.0)	0.554
Hematoma evacuation	18 (5.6)	7 (3.3)	11 (10.1)	0.013
Intensive care unit care	26 (8.2)	20 (9.5)	6 (5.5)	0.213
Clinical outcome				
In-hospital mortality	2 (0.6)	2 (1.0)	0 (0.0)	0.549
Days of hospitalization	2.0 (1.0-4.0)	2.0 (1.0-3.0)	2.0 (1.0-4.0)	0.065

Values are expressed as the median (interquartile range) or as a number (%).
INR, international normalized ratio; pRBCs, packed red blood cells; ED, emergency department; FFP, fresh frozen

plasma; PCs, platelet concentrates.
